# Supplementary material for: Anthropometric and physical fitness indicators in the combine draft between the finalist and the eliminated player in the national basketball association all-star slam dunk contest
Source: PLoS One. 2024 Mar 1;19(3):e0299262. doi: 10.1371/journal.pone.0299262 (PMC10906826; doi:10.1371/journal.pone.0299262)
Supplement: Attachment — Submitted filename: Response to Reviewers.docx [file pone.0299262.s003.docx]

Dear Editor and Reviewers:

Thank you for your letter and for the reviewers’ comments concerning our manuscript entitled “**Anthropometric and physical fitness indicators in the combine draft between the finalist and the eliminated player in the national basketball association all-star slam dunk contest**” (Manuscript Number: **PONE-D-23-19002**). Those comments are all valuable and very helpful for revising and improving our paper, as well as the important guiding significance to our research. We have studied comments carefully and have made corrections which we hope to meet with approval. We highlighted **all revision** in **red** color. The corrections in the paper and the responds to the referee’s comments are as following:

**Additional Editor Comments：**

**Comment 1.** The authors answered almost all the questions made by reviewers. While one reviewer already recommended acception, the other still found some issues in materials and methods.

**OUR REPLY:** Thank for helpful **comments. We have tried best to revise this manuscript. However, we have changed affiliation of the first author with the permission of the editor in Plos One. The reason is that the first author already have enrolled in** Harbin Institute of Technology (HIT) as Phd student, and left previous **affiliation.**

**Comment 2.** In addition, it is suggested replacing average by mean (see tables).

**OUR REPLY:** Thank for your helpful **comments. According to your suggestion, we have** replaced “average” by “mean” in Table 1 and Table 4. Our revision is following:

**Comment 3.** P-values should use the same number of decimal (three). Please standardize them.

**OUR REPLY:** Thank for your helpful **comments.** Based on you**r** suggestion, we have amended them to same number of decimal. As follows:

**Reviewer#1:**

**Comment 1.**First of all, the reviewer would like to thank the authors for their work and efforts in trying to improve sports science knowledge. Overall, the study is well designed and well-written, with a great original article evaluating the usefulness of the
topic

**OUR REPLY:** Thank for your encouragement. We have tried our best to revise the manuscript, aiming to meet the requirement of Plos One.

**Reviewer#3:**

**Comment 1.** The authors answered all the questions and made a considerable effort to follow all the suggestions for improvement. The current version of the manuscript is clearer and more complete than the previous one, and I congratulate the authors on their work. Nevertheless, there are one aspect that still need improvement or clarification, which I'll explain below.

**OUR REPLY:** Thank for your encouragement. We have spared no effort to amend this manuscript to meet the requirement of Plos One.

**Comment 2.** Materials and methods:

Anthropometric Indicators - Unfortunately the information on the protocol used is still missing. The authors have identified a link to the protocols, but when I click on the link I can't identify it. All the questions I raised in Round 1 about the validity of anthropometric data remain. Extracting data from the same database doesn't fully guarantee that:

- the same protocol has always been followed,
- the data was collected by certified anthropometrists.
These issues represent clear threats to the internal validity of the study and should, at the very least, be clearly presented and discussed in the limitations section/paragraph.

**OUR REPLY:** Thank for your helpful comments. We have tried our best to search any useful information that can answer this question from various sources. Unfortunately, we failed to seek out the detailed protocol and test staff’s personal information for NBA combine draft. At present, we can not directly contact the NBA manager, who was in charge of NBA combine draft, for more information. Therefore, We deemed that it is not possible to confirm with certainty that a uniform protocol was followed by certified anthropometrists. In our future study, the standard and detailed protocol was designed and followed by certified anthropometrists for internal validity.

According to your suggestions, we have added one section in the limitations paragraph and ranked it first, clearly discussing this aspect. As follows:

However, this study still has the following limitations. First, although the anthropometric data in NBA combine draft proposes valuable insights, there were certain variations in test protocol and anthropometrist’s qualification, which restricted the internal validity of this study. Second, some NBA draft indicators was excluded, due to the large number of missing value [4]. These indicators were not discussed in this study. **Besides**, the data in this study was **anthropometry and physical fitness test in the draft year, which was not the physical condition during slam dunk contest, so sports injury or other disease may occur in the season after draft** [4]**. However, RSI, exhibiting good reliability on the assessment of jump performance for male basketball players, was not used in the physical fitness test, and it will be used in the future study [33]. Finally, the** number of subjects was comparatively low in the study, because only 3-6 rookies were allowed to join the NBA all star slam dunk contest every year [34]. In the future research, members from slam dunk clubs in the world, such as Team Flight Brother (TFB) in USA, Dunk Elite (DE) in Europe, AirChina (AC) and Dunk King (DK) in China, were recruited as the subjects.
